# Using the 6RL^Ku^ Minichromosome of Rye (*Secale cereale* L.) to Create Wheat-Rye 6D/6RL^Ku^ Small Segment Translocation Lines with Powdery Mildew Resistance

**DOI:** 10.3390/ijms19123933

**Published:** 2018-12-07

**Authors:** Haimei Du, Zongxiang Tang, Qiong Duan, Shuyao Tang, Shulan Fu

**Affiliations:** 1 College of Agronomy, Sichuan Agricultural University, Wenjiang, Chengdu 611130, China; 18314441196@163.com (H.D.); zxtang@sicau.edu.cn (Z.T.); 18380443767@163.com (Q.D.); tangshuyao705708@sina.com (S.T.); 2 Institute of Ecological Agriculture, Sichuan Agricultural University, Wenjiang, Chengdu 611130, China

**Keywords:** wheat, rye, 6R, small segment translocation, powdery mildew

## Abstract

Long arms of rye (*Secale cereale* L.) chromosome 6 (6RL) carry powdery mildew resistance genes. However, these sources of resistance have not yet been successfully used in commercial wheat cultivars. The development of small segment translocation chromosomes carrying resistance may result in lines carrying the 6R chromosome becoming more commercially acceptable. However, no wheat-rye 6RL small segment translocation line with powdery mildew resistance has been reported. In this study, a wheat-rye 6RL^Ku^ minichromosome addition line with powdery mildew resistance was identified, and this minichromosome was derived from the segment between L2.5 and L2.8 of the 6RL^Ku^ chromosome arm. Following irradiation, the 6RL^Ku^ minichromosome divided into two smaller segments, named 6RL^Kumi200^ and 6RL^Kumi119^, and these fragments participated in the formation of wheat-rye small segment translocation chromosomes 6DS/6RL^Kumi200^ and 6DL/6RL^Kumi119^, respectively. The powdery mildew resistance gene was found to be located on the 6RL^Kumi119^ segment. Sixteen 6RL^Kumi119^-specific markers were developed, and their products were cloned and sequenced. Nucleotide BLAST searches indicated that 14 of the 16 sequences had 91–100% similarity with nine scaffolds derived from 6R chromosome of *S. cereale* L. Lo7. The small segment translocation chromosome 6DL/6RL^Kumi119^ makes the practical utilization in agriculture of powdery mildew resistance gene on 6RL^Ku^ more likely. The nine scaffolds are useful for further studying the structure and function of this small segment.

## 1. Introduction

It has already been reported that the long arms of rye (*Secale cereale* L.) chromosome 6 (6RL) carry powdery mildew resistance gene *Pm20* [1], and this gene was introduced into wheat background in the form of a 6BS/6RL translocation chromosome [1]. The gene *Pm20* still has a broad spectrum of resistance to *Blumeria graminis* f. sp. *tritici* (Bgt) isolates [2,3]. The 6RL chromosome arm that carries *Pm20* was derived from *S. cereale* L. cv. Prolific [1]. Recently, some reports indicated that 6RL arms derived from *S. cereale* cv. Jingzhouheimai, *S. cereale* cv. German White, and *S. cereale* cv. Kustro also carried powdery mildew resistance genes [3,4,5,6]. It has already been established that the powdery mildew resistance gene on 6RL of German White was different from the gene *Pm20* [3], and this indicates that different 6RL arms may also display genetic diversity for powdery mildew resistance genes. However, the powdery mildew resistance genes on 6RL arms have not been successfully used in commercial wheat cultivars because of agronomic disadvantages, possibly caused by non-compensation and linkage drag of the 6RL arm. The development of small segment translocations between wheat chromosomes and 6RL, which causes minimal loss of indispensable wheat genes, may resolve this problem [3,4,7,8]. Only one translocation chromosome carrying a small segment of a 6RL arm, Ti4AS.4AL-6RL-4AL, has been reported. In this case, the 6RL segment was derived from the telomeric region and carried the Hessian fly-resistant gene *H25* [9,10]. So far, no wheat-rye 6RL small segment translocation lines with powdery mildew resistance have been reported. In this study, a wheat-rye 6RL^Ku^ minichromosome addition line was developed, and 6D/6RL^Ku^ small segment translocation lines were identified from the irradiated seeds of this minichromosome addition line.

## 2. Results

### 2.1. Obtaining Wheat-Rye 6RL^Ku^ Minichromosome Addition Line

**Figure 1:**
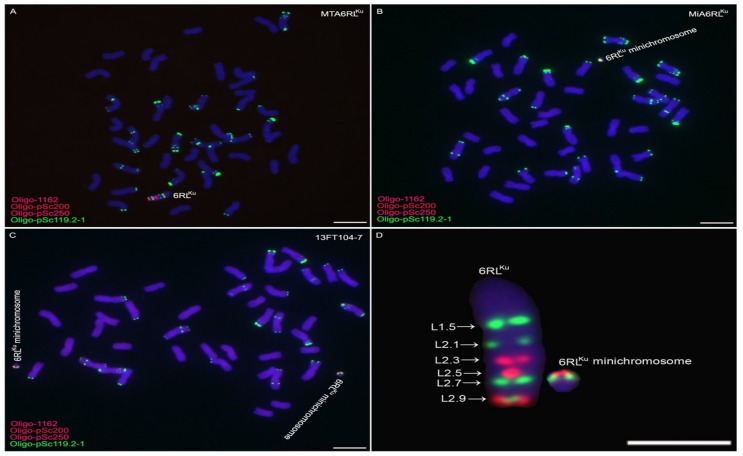
ND-FISH analysis of MTA6RL^Ku^ and MiA6RL^Ku^. (**A**,**B**,**C)** Oligo-1162 (**red**), Oligo-pSc200 (**red**), Oligo-pSc250 (**red**), and Oligo-pSc119.2-1 (**green**) were used as probes. (**D**) Cut and pasted 6RL^Ku^ from MTA6RL^Ku^ and 6RL^Ku^ minichromosome from 13FT104-7. The FISH map of 6RL^Ku^ is the same as the one reported by Li et al. [6]. Chromosomes were counterstained with 4’-6-diamidino-2-phenyllindole (DAPI) (**blue**). Scale bar 10 μm.

A 6RL^Ku^ minichromosome addition line, MiA6RL^Ku^, was found among the self-pollinated progeny of wheat-rye 6RL^Ku^ monotelosomic addition line MTA6RL^Ku^. Line MiA6RL^Ku^ contained one minichromosome derived from the 6RL^Ku^ arm (Figure 1). According to the FISH map of 6RL^Ku^ arm constructed based on the signal patterns of probes Oligo-pSc200, Oligo-pSc250, and Oligo-pSc119.2-1 [6], the 6RL^Ku^ minichromosome was derived from the segment between L2.5 and L2.8 (Figure 1). Through measuring the fraction length of the 6RL^Ku^ arm, combined with the fraction length standard of 6RL built by Mukai et al. [11], it can be deduced that the 6RL^Ku^ minichromosome comprised about 11% of the original 6RL^Ku^ length.

### 2.2. Transmission of 6RL^Ku^ Minichromosome

Thirty-four seeds were randomly selected from the self-fertilized progeny of line MiA6RL^Ku^ for ND-FISH analysis. Among the 34 plants, 24 had no 6RL^Ku^ minichromosome; nine contained one 6RL^Ku^ minichromosome; and one plant, 13FT104-7, contained a pair of this minichromosome (Figure 1C). From the self-fertilized progeny of line 13FT104-7, 100 seeds were randomly selected for ND-FISH analysis; 25 plants contained two 6RL^Ku^ minichromosomes, 62 plants contained one 6RL^Ku^ minichromosomes, and the remaining 13 plants had none of this minichromosome. The progeny of 13FT104-7 were named 15T154, and some of these plants were used for developing additional 6RL^Ku^ minichromosome-specific markers and producing wheat-rye small segment translocations.

### 2.3. Development of 6D/6RL^Ku^ Small Segment Translocation Lines

**Figure 2:**
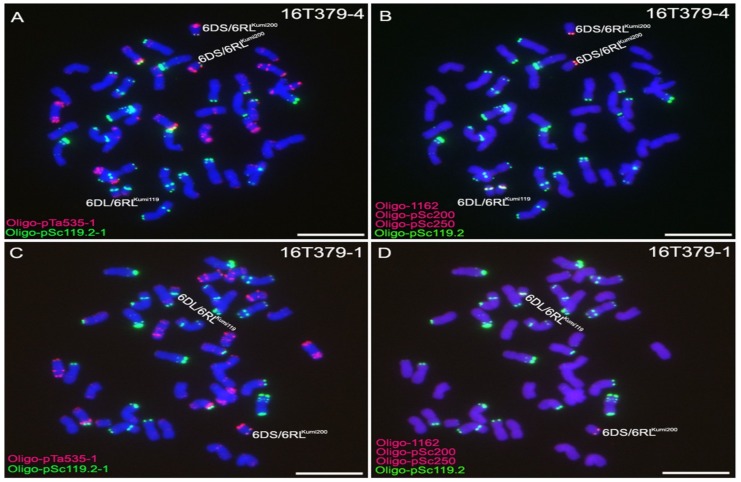
Oligo-pTa535-1 (**red**), Oligo-pSc119.2-1 (**green**), Oligo-1162 (**red**), Oligo-pSc200 (**red**), and Oligo-pSc250 (**red**) were used as probes for ND-FISH analysis of wheat-rye 6D/6RL^Ku^ small segment translocation lines. (**A**,**B**) Line 16T379-4 represents lines with two 6DS/6RL^Kumi200^ and two 6DL/6RL^Kumi119^ chromosomes. (**A**) and (**B**) are the same cell. (**C**,**D**) Line 16T379-1 represents lines with one 6DS/6RL^Kumi200^ and one 6DL/6RL^Kumi119^ chromosome. (**C**) and (**D**) are the same cell. Chromosomes were counterstained with DAPI (**blue**). Scale bar 10 μm.

Some seeds that were derived from line 14T154-35 with two 6RL^Ku^ minichromosomes were exposed to ^60^Co-γ rays. A total of 1428 M1 seeds were analyzed using ND-FISH, and ten wheat-rye 6D/6RL^Ku^ small segment translocation lines were detected and named 16T379 or 16T380 (Table 1, Figure 2). In these 6D/6RL^Ku^ small segment translocation lines, the 6RL^Ku^ minichromosome divided into two smaller segments. One small segment, with the Oligo-pSc200 and Oligo-pSc250 signals and a strong Oligo-pSc119.2-1 signal, was named 6RL^Kumi119^. The other small segment, with the Oligo-pSc200 and Oligo-pSc250 signals and a weak Oligo-pSc119.2-1 signal, was named 6RL^Kumi200^. The segment 6RL^Kumi200^ had been translocated onto the 6DS arm, while the segment 6RL^Kumi119^ was translocated onto the 6DL arm (Figure 2). The small segment translocation chromosome fused with 6DS was named 6DS/6RL^Kumi200^, and the other translocation attached with 6DL was named 6DL/6RL^Kumi119^ (Figure 2). 

### 2.4. Transmission Rates of 6DS/6RL^Kumi200^ and 6DL/6RL^Kumi119^

**Figure 3:**
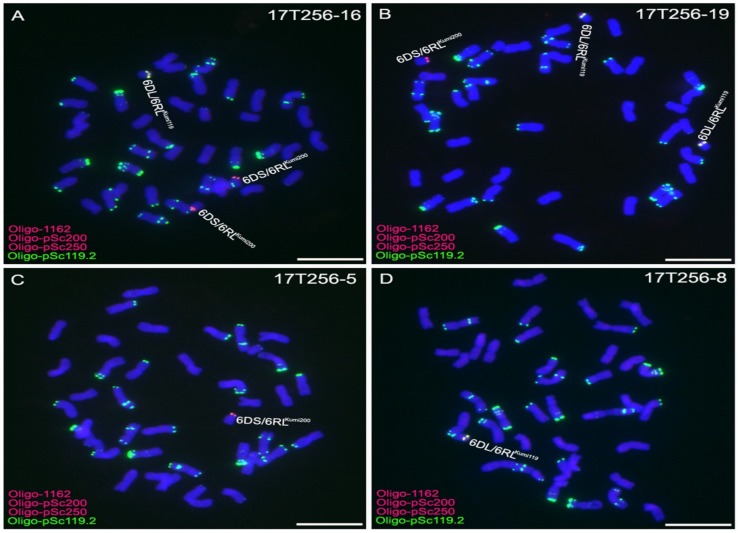
Oligo-pSc119.2-1 (**green**), Oligo-1162 (**red**), Oligo-pSc200 (**red**), and Oligo-pSc250 (**red**) were used as probes for ND-FISH analysis of the progeny of line 16T379-1. (**A**) Line 17T256-16 carried two 6DS/6RL^Kumi200^ chromosomes and one 6DL/6RL^Kumi119^ chromosome. (**B**) Line 17T256-19 carried one 6DS/6RL^Kumi200^ and two 6DL/6RL^Kumi119^ chromosomes. (**C**) Line 17T256-5 possessed only one 6DS/6RL^Kumi200^ chromosome. (**D**) Line 17T256-8 had only one 6DL/6RL^Kumi119^ chromosome. Chromosomes were counterstained with DAPI (**blue**). Scale bar 10μm.

Among 100 randomly selected seeds from the progeny of 16T379-1, 21 derived seedlings contained no translocation chromosomes, 16 seedlings contained two 6DS/6RL^Kumi200^ and two 6DL/6RL^Kumi119^ chromosomes, 42 seedlings contained one 6DS/6RL^Kumi200^ and one 6DL/6RL^Kumi119^ chromosome, eight contained two 6DS/6RL^Kumi200^ and one 6DL/6RL^Kumi119^ chromosome (Figure 3A), three plants contained one 6DS/6RL^Kumi200^ and two 6DL/6RL^Kumi119^ chromosomes (Figure 3B), three plants contained only one 6DS/6RL^Kumi200^ chromosome (Figure 3C), and seven plants contained only one 6DL/6RL^Kumi119^ chromosome (Figure 3D). The progeny of 16T379-1 were named 17T256, and some of these plants were used for further marker development. All of the randomly selected 100 seeds from the progeny of 16T379-4 contained two 6DS/6RL^Kumi200^ and two 6DL/6RL^Kumi119^ chromosomes. These results indicate that the 6DS/6RL^Kumi200^ and 6DL/6RL^Kumi119^ chromosomes show a high frequency of transmission to progeny.

### 2.5. Development of 6RL^Ku^ Minichromosome-Specific Markers

**Figure 4:**
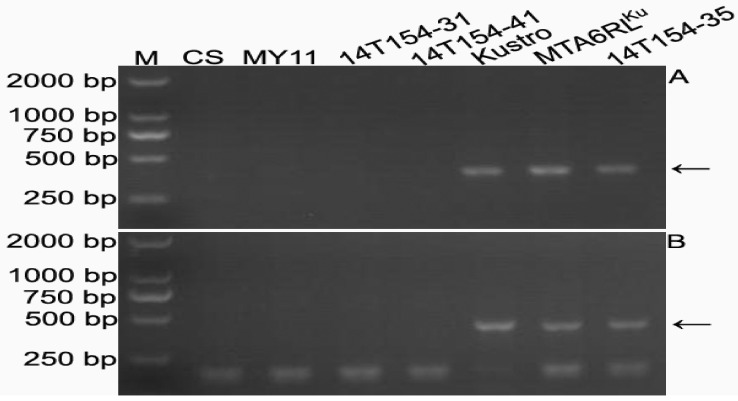
Representative results of developing 6RL^Ku^ minichromosome-specific markers. (**A**) Products amplified marker 6RL-M55. (**B**) Products amplified marker 6RL-M63. M, DNA marker. Arrows indicate 6RL^Ku^ minichromosome-specific bands.

**Figure 5:**
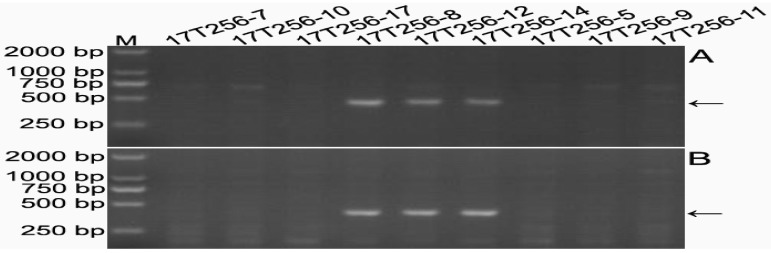
Representative results of locating 6RL^Ku^ minichromosome-specific markers on 6RL^Kumi119^ segment. (**A**) Products amplified by marker 6RL-M11. (**B**) Products amplified by marker 6RL-M102. M, DNA marker. Arrows indicate 6RL^Kumi119^-specific bands.

Common wheat Chinese Spring (CS), Mianyang 11 (MY11), rye Kustro, line MTA6RL^Ku^, line 14T154-35 with two 6RL^Ku^ minichromosomes, and two lines (14T154-31 and 14T154-41) without the minichromosome were used to develop 6RL^Ku^ minichromosome-specific markers. One thousand and eighty-six pairs of primers were designed according to the 6RL^Ku^ minichromosome-specific pair-end reads. Sixteen of the 1086 primer pairs amplified their target bands from the genomic DNAs of rye Kustro, line MTA6RL^Ku^, and line 14T154-35, and not from the genomic DNA of CS, MY11, and lines 14T154-31 and 14T154-41 (Figure 4). Therefore, the 16 primer pairs were tentatively regarded as 6RL^Ku^ minichromosome-specific markers and are listed in Table 2. From the progeny of 16T379-1, three lines (17T256-7, 17T256-10, and 17T256-17) without 6D/6RL^Ku^ translocation chromosomes, three lines (17T256-8, 17T256-12, and 17T256-14) that only contained one 6DL/6RL^Kumi119^ chromosome, and three lines (17T256-5, 17T256-9, and 17T256-11) that only contained one 6DS/6RL^Kumi200^ chromosome, were used to validate the 16 primer pairs. The 16 primer pairs only amplified target bands from lines 17T256-8, 17T256-12, and 17T256-14 (Figure 5). This indicated that all the 16 6RL^Ku^ minichromosome-specific markers were located on the segment 6RL^Kumi119^. Therefore, the 16 markers were 6RL^Kumi119^-specific.

### 2.6. Sequence Characteristics of the Products Amplified by the 16 Markers

The sequences amplified by the 16 6RL^Kumi119^-specific markers were isolated. These sequences have been deposited in the GenBank Database (GenBank accession numbers: MK051036–MK051051). Sequence alignment using the BLAST tool in NCBI and gene annotation indicated that all the 16 sequences did not belong to any gene sequence; therefore, they are probably not involved in active genes. Analysis using RepeatMasker software (http://repeatmasker.org/) also indicated that these sequences were not involved in repetitive DNA sequences. Nucleotide BLAST searches indicated that 14 of the 16 sequences had 91–100% similarity with the scaffolds from the 6R chromosome of *S. cereale* L. Lo7, and the other two sequences had 99–100% similarity with the scaffolds derived from 0R of *S. cereale* L. Lo7 scaffolds (Table 2). It can be noted that all the five sequences (MK051040, MK051042, MK051044, MK051045, and MK051047) were located in the scaffold Lo7_v2_scaffold_445202 6R, and both MK051050 and MK051051 were located in the scaffold Lo7_v2_scaffold_445253 6R (Table 2). According to the gene annotation of these scaffolds published by Bauer et al. [12], they did not contain genes; however, these scaffolds were still useful for the future study on the structure and function of the 6RL^Kumi119^ segment.

### 2.7. Powdery Mildew Resistance

**Figure 6:**
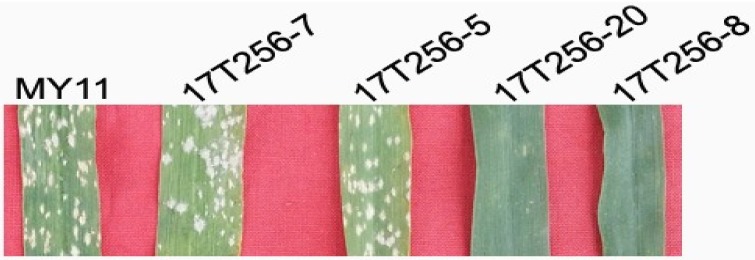
Identification of resistance to powdery mildew. Parental wheat and lines without 6D/6RL^Ku^ translocation chromosomes and lines with only one 6DS/6RL^Kumi200^ chromosome are highly susceptible to powdery mildew. Lines with one 6DS/6RL^Kumi200^ and one 6DL/6RL^Kumi119^ chromosome and lines with only one 6DL/6RL^Kumi119^ chromosome are highly resistant to powdery mildew.

Powdery mildew resistance testing indicated that parental wheat MY11, lines without 6RL^Ku^ minichromosome, and lines with only one 6DS/6RL^Kumi200^ chromosome were highly susceptible to powdery mildew (Figure 6). Lines with both the 6DS/6RL^Kumi200^ and 6DL/6RL^Kumi119^ chromosomes and lines with only one 6DL/6RL^Kumi119^ chromosome displayed high resistance to powdery mildew (Figure 6). These results indicated that the powdery mildew resistance gene on 6RL^Ku^ was located on the small segment 6RL^Kumi119^. 

## 3. Discussion

### 3.1. Extending Genetic Basis of Powdery Mildew Resistance Genes

Several reports have highlighted the current narrowness of genetic diversity of powdery mildew resistance genes in wheat breeding programs in China [13,14,15]. For example, the wheat cultivars (lines) from the Yangtze River region mainly contained genes *Pm4a* and *Pm21* [13]. The gene *Pm21* is widely used in wheat cultivars from Sichuan and Guizhou provinces [14], while the wheat cultivars (lines) from Henan province mainly carry genes *Pm2*, *Pm4*, *Pm21*and unknown gene(s) located on a new 1RS.1BL translocation chromosome [15]. It should be noted that gene *Pm21* has played an important role in wheat powdery mildew resistance breeding programs in China, but there is the risk of pathogen directional selection caused by the high frequency of *Pm21* gene in wheat breeding programs [15]. Therefore, extending the genetic basis of powdery mildew resistance genes in wheat breeding programs in China must be an urgent priority. 6R chromosomes of rye (*S. cereale* L.) carry powdery mildew resistance genes, and still display effective resistance to powdery mildew pathotypes in China [1,2,3,4,5,6,16]. In addition, the powdery mildew resistance genes on 6R chromosomes have genetic diversity [3,16], which may provide recipient wheat lines with resistance to future virulent powdery mildew races that might defeat existing genes. However, these 6R-derived powdery mildew resistance genes have not been successfully used in commercial wheat cultivars because of the loss of indispensable wheat genes and alien chromosome-associated linkage drag. Therefore, the development of new wheat-rye 6RL small segment translocation chromosomes with powdery mildew resistance gene(s) is of pressing concern [3,4,8]. It has been reported that few recombinants (sometime none) can be recovered between 6R and wheat chromosomes, even in a *ph1b* mutant background. Furthermore, it is difficult to produce suitable wheat-rye 6RL small segment translocations [17]. In this study, a wheat-rye small segment translocation chromosome 6DL/6RL^Kumi119^ with powdery mildew resistance was developed using a 6RL^Ku^ minichromosome. According to the fraction length standard of the 6RL arm constructed by Mukai et al. [11], the size of the 6RL^Kumi119^ segment might be slightly larger than the small 6RL segment in the Ti4AS.4AL-6RL-4AL translocation chromosome, in which the 6RL segment occupied 10% of the total length of 6RL [11]. Therefore, the occurrence of possible deleterious genes on the 6RL^Ku^ arm may have been greatly reduced. Additionally, the 6DS/6RL^Kumi200^ and 6DL/6RL^Kumi119^ chromosomes showed high transmission to progeny. These characteristics make the successful utilization of the powdery mildew resistance gene on 6RL^Ku^ more likely, although the agronomic traits of the lines with 6DS/6RL^Kumi200^ and 6DL/6RL^Kumi119^ chromosomes have not been investigated.

### 3.2. 6RL^Ku^ Minichromosome Specific Markers

For the effective utilization of elite genes in wild germplasm, it is necessary to increase our understanding of the molecular basis of target alien chromosome regions [18,19]. In this study, the small segment translocation chromosome 6DL/6RL^Kumi119^ provides an opportunity to understand the molecular basis of the small segment 6RL^Kumi119^ carrying powdery mildew resistance. Additionally, 16 6RL^Ku^ minichromosome-specific markers were developed, and these markers were subsequently located onto the segment 6RL^Kumi119^ with powdery mildew resistance. These markers represent convenient tools to use the 6DL/6RL^Kumi119^ translocation chromosome in wheat breeding programs. Using the sequences amplified by the 16 markers, 11 scaffolds derived from winter rye inbred line *S. cereale* L. Lo7 [12] were located on the segment 6RL^Kumi119^. These scaffolds are useful for the further detailed studying of this segment. However, published data indicated that no active genes could be found among the 11 scaffolds [12]. There are two possible explanations for this phenomenon. First, the 6RL^Ku^ minichromosome-specific markers may be insufficient in number. Second, large gaps may exist in these scaffolds. Therefore, a greater number of 6RL^Ku^ minichromosome-specific markers are required and more complete rye genomic sequences are needed.

## 4. Materials and Methods

### 4.1. Plant Materials

A monotelosomic addition line MTA6RL^Ku^ was developed according to the methods described by Qiu et al. [20]. From the self-pollinated progeny of MTA6RL^Ku^, a 6RL^Ku^ minichromosome addition line MiA6RL^Ku^ was identified. Some seeds selected from the progeny of MiA6RL^Ku^ were irradiated with ^60^Co-γ rays at the Biotechnology and Nuclear Technology Research Institute, Sichuan Academy of Agricultural Sciences, China. Common wheat (*Triticum aestivum* L.) Mianyang 11 (MY11) and Chinese Spring (CS) were used as controls.

### 4.2. Cytological Analysis

The root-tip metaphase cells were analyzed using non-denaturing fluorescence *in situ* hybridization (ND-FISH) technology. Oligonucleotide (oligo) probes Oligo-1162, Oligo-pSc200, Oligo-pSc250, Oligo-pSc119.2-1, and Oligo-pTa535-1 [21,22] were used. These oligo probes were 5′end-labelled with 6-carboxyfluorescein (6-FAM) or 6-carboxytetramethylrhodamine (Tamra). Root-tip metaphase chromosomes were prepared following the methods described by Han et al. [23]. ND-FISH analysis was performed following the methods described by Fu et al. [22] with the following minor modification. When dropped onto cell spreads, the probe mixture containing Oligo-1162 around the slides was kept above 28 °C, and the slides were immediately placed into a moist box that was incubated at 42 °C in advance for 1–2 h. Then, the slides were washed 15–20 s in 2 × SSC at 42 °C. 

### 4.3. Development of 6RL^Ku^ Minichromosome-Specific PCR-Based Markers

Genomic DNAs of *S. cereale* L. Kustro and MiA6RL^Ku^ were sequenced by using the Specific Length Amplified Fragment Sequencing (SLAF-seq) technique (Biomarker, Beijing, China). The sequencing procedure followed the methods described by Duan et al. [24]. The pair-end reads derived from Kustro and MiA6RL^Ku^ were compared with the whole genome shotgun assembly sequences (IWGSC WGA v0.4) of common wheat *cv.* Chinese Spring (*Triticum aestivum* L.) (http://www.wheatgenome.org) using SOAP software (Beijing Genomics Institute (BGI), Beijing, China) [25]. The pair-end reads with high wheat homology were discarded. At last, after comparing specific pair-end reads of Kustro and MiA6RL^Ku^, the 6RL^Ku^ minichromosome-specific pair-end reads were obtained. Primers were designed according to the 6RL^Ku^ minichromosome-specific pair-end reads using the software Primer 3 (version 4.0). The optimal melting temperature and size values of primers were set following the methods described by Duan et al. [24]. 

### 4.4. PCR Analysis and Sequence Cloning

The PCR amplifications were carried out according to the procedure described by Li et al. [6]. Agarose gel (2%) electrophoresis was used to detect the amplicons in 1 × TAE buffer. The products amplified by the 6RL^Kumi119^-specific primers were recovered using Universal DNA Purification Kit (Tiangen Biotech Co., Ltd, Beijing, China) and cloned using pClone007 Vector Kit (TsingKe Biotech Co., Ltd, Beijing, China). Inserts were sequenced by TsingKe Biotech (Beijing) Co., Ltd. These sequences were deposited in the GenBank Database. Finally, these sequences were used for Nucleotide BLAST searches against the *S. cereale* L. Lo7 scaffolds database using the BLAST tool in GrainGenes (https://wheat.pw.usda.gov/cgi-bin/seqserve/blast_rye.cgi) and gene annotation was carried out according to the data sets related to the publication by Bauer et al. [12]. Additionally, RepeatMasker software (http://repeatmasker.org/) was used to identify whether these sequences amplified by the 6RL^Kumi119^-specific primers are repetitive DNA.

### 4.5. Powdery Mildew Resistance Test

The resistance of line MiA6RL^Ku^, the progeny of MiA6RL^Ku^, 6D/6RL^Ku^ small segment translocation lines, and parental wheat MY11 to powdery mildew were evaluated. Plants were grown in Qionglai, Sichuan, China. The materials were naturally inoculated by locally occurring field derived powdery mildew, and infection types (IT) were scored according to the standard described by Fu et al. [5].

## 5. Conclusions

In conclusion, a new wheat-rye small segment translocation chromosome 6DL/6RL^Kumi119^ with powdery mildew resistance was produced. Sixteen 6RL^Kumi119^ specific markers were developed, and 11 *S. cereale* L. Lo7 scaffolds were located on the small rye segment 6RL^Kumi119^. The small segment translocation chromosome 6DL/6RL^Kumi119^ makes the practical utilization of the powdery mildew resistance gene on 6RL^Ku^ more likely. The 6RL^Kumi119^-specific markers and the *S. cereale* L. Lo7 scaffolds that were located on the 6RL^Kumi119^ segment may find application in future detailed studies of the structure and function of this small segment.

## Figures and Tables

**Table 1:** Wheat-rye 6D/6RL^Ku^ small segment translocation lines with 6DS/6RL^Kumi200^ and 6DL/6RL^Kumi119^ translocation chromosomes.

Small Segment Translocation Lines	Small Segment Translocation Chromosomes
16T379-1	one 6DS/6RL^Kumi200^ and one 6DL/6RL**^Kumi119^**
16T379-4	two 6DS/6RL^Kumi200^ and two 6DL/6RL**^Kumi119^**
16T379-6	one 6DS/6RL^Kumi200^ and one 6DL/6RL^Kumi119^
16T379-8	two 6DS/6RL^Kumi200^ and two 6DL/6RL**^Kumi119^**
16T379-9	one 6DS/6RL^Kumi200^ and one 6DL/6RL**^Kumi119^**
16T379-11	one 6DS/6RL^Kumi200^ and one 6DL/6RL**^Kumi119^**
16T379-13	one 6DS/6RL^Kumi200^ and one 6DL/6RL**^Kumi119^**
16T379-14	two 6DS/6RL^Kumi200^ and two 6DL/6RL**^Kumi119^**
16T380-2	one 6DS/6RL^Kumi200^ and one 6DL/6RL**^Kumi119^**
16T380-3	one 6DS/6RL^Kumi200^ and one 6DL/6RL**^Kumi119^**

**Table 2:** 6RL^Ku^ minichromosome-specific markers and the similarity between the sequences amplified by the markers and the *S. cereale* Lo7 scaffolds.

Marker	Forward (5′-3′)	Reverse (5′-3′)	GenBank Accession Number of Amplified Sequence	Similarity of Amplified Sequence with the *S. cereale* Lo7 Scaffolds
6RL-M8	CAACCTATTCGGACCAGAGC	GATTAAACCGCTGGTGAGAAAC	MK051036	99% similarity with 7794–8206 bp of Lo7_v2_scaffold_453717 6R
6RL-M11	GGGGGAACTTTGAGTATGCTT	GATCGGATCGGTTGAGTTGT	MK051037	99% similarity with 464–1239 bp of Lo7_v2_scaffold_651086 0R
6RL-M55	TGATGCAAGTTCGTTGGTGT	CGTTGACTCCCTTCCGTTAG	MK051038	91% similarity with 1–108 bp of Lo7_v2_scaffold_457844 6R
6RL-M63	TCGAAATGCATCGGACAAT	TCCATGGTCTCCTCGAGTGT	MK051039	100% similarity with 144–422 bp of Lo7_v2_scaffold_492428 6R
6RL-M102	CGGGAGAGGACTGGTTCTT	CATATGTACAACAGAGGCATCTTC	MK051040	98% similarity with 26957–27168 bp, 27841-27881 bp and 27923-28047 bp of Lo7_v2_scaffold_445202 6R
6RL-M118	TCCCCCTTCTAGGGTTTCAT	ATAGCCCCATCTGCAAACAC	MK051041	100% similarity with 488–896 bp of Lo7_v2_scaffold_484582 6R
6RL-M149	AATGGCTGCAATTTCTTGGA	AAAAAGCCACAAAACACTGC	MK051042	100% similarity with 34805–34422 bp of Lo7_v2_scaffold_445202 6R
6RL-M220	GCACAAGTCCATGTCCTTCA	GATCCATCTGGCTGTGTGTG	MK051043	99% similarity with 4112–4449 bp of Lo7_v2_scaffold_448816 6R
6RL-M221	CGCTATATGCAATGCAGGTG	CTTGCTTGCAACACCAAAAA	MK051044	98% similarity with 41511–41912 bp of Lo7_v2_scaffold_445202 6R
6RL-M255	CCTTATGACCACCCATGCTC	TTCATAGCTGCCTCTTTTAGGTG	MK051045	99% similarity with 31812–32230 bp of Lo7_v2_scaffold_445202 6R
6RL-M710	CAAACTCACACGAAGCCAAA	CTGATCCAAATTTGCCCAGT	MK051046	92% similarity with 3600–3681 bp of Lo7_v2_scaffold_457146 6R
6RL-M828	TTTGTCGAGAGCAACAATGG	CCCGCTTCTAAGTTCAATCG	MK051047	100% similarity with 39318–39668 bp of Lo7_v2_scaffold_445202 6R
6RL-M869	GGGTCAACCCATCTTGTTTC	CCTCTTCCACTGCAGAGCTT	MK051048	99% similarity with 589–962 bp of Lo7_v2_scaffold_451612 6R
6RL-M896	GACGAAACACAACAAATCATTCA	GGGAAAATCGAAAACTGCAA	MK051049	100% similarity with 10161–10344 bp of Lo7_v2_scaffold_620512 0R
6RL-M1074	AAAGCCGATGAAAAATGGTG	GAAGAAGAAGAAGATGGGGTGTT	MK051050	100% similarity with 9159–9365 bp of Lo7_v2_scaffold_445253 6R
6RL-M1081	TTGCATGCTCGCTTTAGTTG	CCACTTGACGTTGCCCTATT	MK051051	100% similarity with 8940–9193 bp of Lo7_v2_scaffold_445253 6R
